# Own-price and cross-price elasticities of demand for cigarettes and waterpipe tobacco in three Eastern Mediterranean countries: a volumetric choice experiment

**DOI:** 10.1136/tobaccocontrol-2021-056616

**Published:** 2021-06-30

**Authors:** Ali Chalak, Rima Nakkash, Niveen M E Abu-Rmeileh, Yousef S Khader, Mohammed Jawad, Aya Mostafa, Ruba Abla, Jordan Louviere, Ramzi G Salloum

**Affiliations:** 1 Department of Agriculture, Faculty of Agricultural and Food Sciences, American University of Beirut, Beirut, Lebanon; 2 Department of Health Promotion and Community Health, Faculty of Health Sciences, American University of Beirut, Beirut, Lebanon; 3 Institute of Community and Public Health, Birzeit University, Birzeit, Palestine; 4 Department of Community Medicine, Public Health and Family Medicine, Faculty of Medicine, Jordan University of Science and Technology, Irbid, Jordan; 5 Public Health Policy Evaluation Unit, Imperial College London, London, UK; 6 Department of Community, Environmental, and Occupational Medicine, Faculty of Medicine, Faculty of Medicine, Ain Shams University, Cairo, Egypt; 7 Choiceflows, LLC, Chapel Hill, North Carolina, USA; 8 Department of Health Outcomes and Biomedical Informatics, College of Medicine, University of Florida, Gainesville, Florida, USA

**Keywords:** economics, taxation, low/middle income country, global health, non-cigarette tobacco products

## Abstract

**Background:**

Waterpipe tobacco smoking rates in the Eastern Mediterranean region are among the highest worldwide, yet little evidence exists on its economics. Estimates of demand elasticities for tobacco products are largely limited to cigarettes. This study aimed to estimate own-price and cross-price elasticities of demand for cigarettes and waterpipe tobacco products in Lebanon, Jordan and Palestine.

**Methods:**

A volumetric choice experiment was conducted using nationally representative household surveys. The choice experiment elicited respondents’ stated purchases of eight cigarette and waterpipe tobacco product varieties by hypothetically varying prices. Data were analysed using zero-inflated Poisson models that yielded demand elasticity estimates of cigarette and waterpipe tobacco consumption.

**Results:**

The study included 1680 participants in Lebanon (50% female), 1925 in Jordan (44.6% female) and 1679 in Palestine (50% female). We found the demand for premium cigarettes to be price elastic (range, −1.0 to −1.2) across all three countries, whereas the demand for discount cigarettes was less elastic than premium cigarettes in Lebanon (−0.6) and Jordan (−0.7) and more elastic in Palestine (−1.2). The demand for premium waterpipe tobacco was highly elastic in Lebanon (−1.9), moderately elastic in Jordan (−0.6) and inelastic in Palestine (0.2). The cross-price elasticity between cigarettes and waterpipe tobacco was near zero, suggesting that the two products are not considered to be close substitutes by consumers.

**Conclusions:**

These results serve as a strong evidence base for developing and implementing fiscal policies for tobacco control in the Eastern Mediterranean region that address cigarettes and waterpipe tobacco products.

## Introduction

Tobacco use is the leading cause of preventable mortality globally, with nearly 1 billion deaths projected for the 21st century.^1^ The majority of the world’s smokers live in low/middle-income countries (LMICs), which are expected to be disproportionately impacted by the adverse consequences of tobacco use this century.^2^ The WHO has called attention to the ‘vicious circle’ of tobacco and poverty, recognising that the death, disease, loss of income and loss of productivity due to tobacco use all contribute to poverty, along with the diversion of household funds from necessary resources, such as food, shelter and education, to tobacco purchases.^3^ A growing body of evidence globally, including that from LMICs, clearly demonstrate that tobacco taxes are a powerful tool for reducing tobacco use while providing a reliable source of government revenues.^4^

The Eastern Mediterranean region has the lowest average prices of tobacco products among all WHO regions,^5^ and is the only region for which smoking prevalence has been projected to increase by 2025.^6^ Tobacco product prices are an important factor when considering that decreasing affordability is the most effective strategy to reduce the uptake of smoking among young people.^7^ In addition to the public health toll of cigarette smoking, there is a growing concern about the increased prevalence of waterpipe smoking in the region.^8^ The WHO Framework Convention on Tobacco Control recommends that taxation policy take into account price elasticity of demand to reduce tobacco consumption, and that all tobacco products be taxed comparably to avoid unintended consequences, such as product substitution.^6^

Despite the growing concern over tobacco use in LMICs across the Eastern Mediterranean and other regions, evidence on the economic determinants of smoking has mostly been limited to cigarette smoking and concentrated in high-income countries.^9^ Given that waterpipe smoking prevalence is on par with cigarette smoking prevalence across the region, there is a need for economic research that more accurately captures the unique context of waterpipe smoking.

Research from Lebanon has examined the effect of taxation on tobacco consumption and public revenues using the 2005 national survey of household living conditions, estimating the own-price elasticity for local cigarettes (−1.5), imported cigarettes (−0.2) and waterpipe tobacco (−1.4), as well as the cross-price elasticity between waterpipe tobacco and locally produced cigarettes (0.1) and imported cigarettes (0.2).^10^ The researchers projected that increasing taxes on all tobacco products so as to double the price of imported cigarettes would increase government revenues by 75%, and that a 50% increase in the price of tobacco through excise taxes would lead to an estimated 65 000 premature deaths averted and $300 million in additional tax revenues.^11^ However, one limitation of the study was that the data were collected at the household level, and the researchers were unable to account for individual smokers within the household. Further, the expenditure data captured waterpipe tobacco consumption at home but not at restaurants or cafés. Given that a significant portion of waterpipe tobacco smokers in the region smoke exclusively at commercial establishments,^12–14^ many waterpipe smokers may have been excluded in these estimates. In Jordan, the price elasticity of demand for cigarette smoking was estimated to be −0.6 using national survey data from 2011, whereby smoking among men was more responsive to price than among women (elasticity of −0.8).^15^ However, the study did not model the impact of waterpipe tobacco price increases.

Demand elasticities are key parameters, not only to evaluate the potential effects of taxes on tobacco consumption, but also to estimate revenue, understand the potential differences between price elasticities and the effects of a tax, and to predict substitution among different tobacco products in response to price increases. A recent systematic review and meta-analysis focusing on the elasticity of demand for non-cigarette tobacco products found sufficient evidence in support of the effectiveness of price increases to reduce consumption of non-cigarette tobacco products.^16^ However, the aforementioned study from Lebanon was the only included study that reported price elasticity estimates for waterpipe tobacco.^10^

Given the need for research on the economics of tobacco in the Eastern Mediterranean, especially research on waterpipe tobacco, our objective was to estimate own-price and cross-price elasticities of demand for cigarettes and waterpipe smoking in Lebanon, Jordan and Palestine. Lebanon and Palestine apply mixed excise tax structures, whereas Jordan uses a specific excise tax.^17^ In 2018, total taxes on cigarettes in Lebanon were estimated at 45.6% of the retail price, including 7.1% in excise tax, 28.7% in ad valorem tax, 8.4% in value added tax and 1.3% in import duty. In Jordan, total taxes on cigarettes were estimated at 79.9% of the retail price, including 70.8% in specific excise tax and 9.2% in value added tax; and in Palestine, total taxes on cigarettes were estimated at 83.5%, including 35.9% in specific excise tax, 33.8% in ad valorem tax and 13.8% in value added tax. In 2018, one pack of the most sold brand was priced at the equivalent of $0.76 in Lebanon, $2.39 in Jordan and $6.00 in Palestine, based on official exchange rates between local currency and the US dollar.^17^

The current study evaluated the effects of price differences on the demand for cigarette and waterpipe tobacco products using choice experiments, which are increasingly used in tobacco control research as they allow for the assessment of policy configurations prior to their implementation.^18^ Previous research using choice experiments demonstrated the importance of price for waterpipe smoking demand.^19 20^ In particular, we used volumetric choice experiments, a novel variant that extends choice experiments to allow for assessment of count data in order to mirror real-world decisions that involve the purchase of multiple units of the same product.^21 22^

## Methods

### Study population

Data were collected using nationally representative household surveys of adults (≥18 years old) in Lebanon, Jordan and Palestine between August and November 2019. Study participants were selected using a multistage cluster sampling approach with the probability-proportional-to-size random selection method. In each country, the sample of households was chosen in two stages, by first selecting well-defined geopolitical clusters within each governorate (ie, province) and then selecting housing units within each cluster. One eligible male and one female resident were selected from each selected household. After eligibility screening, we provided potential respondents with information about the study and asked them to provide consent to participate.

### Study procedures

The same methods and recruitment protocol were used in all three countries. Data collectors consented participants before administering the survey, which included the Arabic-language questionnaire and the choice experiment. The questionnaire assessed sociodemographic characteristics (ie, sex, age, education, marital status, employment, education, household income and nationality), as well as cigarette and waterpipe smoking (ie, smoking status, frequency, intention to quit, previous quit attempt(s) and waterpipe smoking locations). The questionnaire was informed by prior, validated surveys of tobacco use^23–25^ and pretested using cognitive interviews to ensure comprehension.

### Experimental protocol

**Figure 1:**
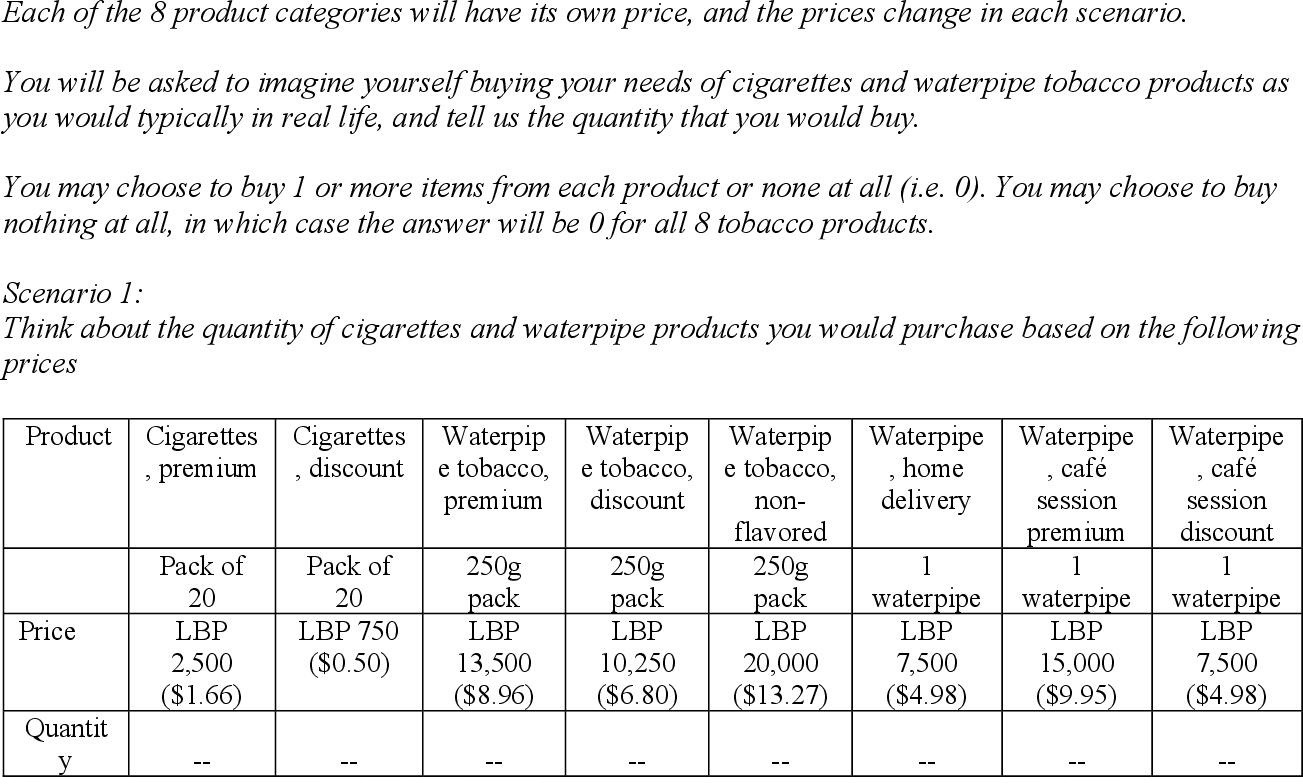
An example of a volumetric choice experiment choice set in Lebanon. This example illustrates one of eight choice sets presented to therespondent in the experiment. Prices for each product varied across choicesets. LBP, Lebanese pounds; US dollar equivalent is calculated based on theexchange rate at the time of the survey (ie, $1=LBP 1507). Thequestionnaire was conducted in Arabic. This illustration represents an Englishtranslation.

Choice experiments offer an advantage over non-experimental purchase data in avoiding endogeneity problems through randomisation of attribute levels and well-defined choice sets.^26^ By simultaneously offering multiple distinct volumetric choices among competing products, volumetric choice experiments allow for the estimation of a complete set of own-price and cross-price elasticities without the need to restrict the relationship between those elasticities. The current study modelled eight-way, mutually exclusive choices among: (1) premium cigarettes, (2) discount cigarettes, (3) premium waterpipe tobacco, (4) discount waterpipe tobacco, (5) non-flavoured waterpipe tobacco, (6) waterpipe tobacco home delivery, (7) premium café waterpipe smoking session and (8) discount café waterpipe smoking session. Products (3)–(5) were presented as packaged waterpipe tobacco for home consumption, whereas product (6) was presented as ready-to-smoke waterpipe delivered to the home. Flavoured cigarettes are not common in these countries, and therefore, cigarettes were presented as non-flavoured. However, flavoured waterpipe is dominant, and therefore, all waterpipe products were presented as flavoured except when indicated. In Palestine, waterpipe home delivery is not available, and was replaced with roll-your-own cigarettes as a product commonly consumed in that market. Each product varied according to four price levels, with the base price reflecting current average market prices in each country, and each subsequent level reflecting a 50% incremental increase (figure 1). The experimental design generated 32 possible product combinations; however, to reduce response burden, participants were randomised to evaluate one of four blocks of eight choice sets. For each choice set, participants were asked to state the quantity of cigarettes and waterpipe products they would purchase based on the prices of each product. Respondents could choose one or more of each product or none at all, depending on their preferences.

### conometric analysis

Participant characteristics were analysed using summary statistics, testing for pairwise country differences in current smoking using the two-sample proportion tests. The volumetric choice experiments were analysed using zero-inflated Poisson regression, used to model count data that have an excess of zero counts (ie, non-users of tobacco products), and whose key property is that the conditional mean is constrained to be equal to the conditional variance.^27^ We followed best practice for estimating elasticity of demand for smoking by including the entire population, rather than smokers only.^28^ This approach has the added advantage of simultaneously explaining the likelihood of smoking, in addition to the quantity smoked conditional on price. Thus, the models had two parts—a Poisson count model and the logit model for predicting excess zeros. In total, 24 models were fitted—eight in each country corresponding to the products under examination. The primary aim of the zero-inflated models was to estimate own-price and cross-price elasticities by including in each of the product-specific count models all product prices that appeared in the choice set. As the price of any tobacco product *i* was included in logarithmic form, its coefficient *ε_ij_* in the count model for any product *j* was therefore an estimate of the cross-price elasticity measuring the responsiveness of demand for product *j* to a change in the price of product *i*. When *i* and *j* were identical, the elasticity measure represented the own-price elasticity of that product. The count models also controlled for income effects by means of indicator variables representing various income groups. The logit models included additional covariates—sex, age, marital status, employment, nationality and governorate, in addition to the respondent’s current cigarette smoking status and waterpipe smoking status, and their interactions.

## Results

### Sample characteristics

The survey was completed by a total of 1680 respondents (50% female) in Lebanon, 1925 respondents (44.6% female) in Jordan and 1679 respondents (50% female) in Palestine (table 1). Age distribution exhibited similar patterns across all three countries, with nearly two-thirds of respondents aged 45 years or younger (62.4%, 64.2% and 62.7% in Lebanon, Jordan and Palestine, respectively). Compared with the other two countries, Lebanon had a higher proportion of full-time employment (44.5% compared with 34.4% in Jordan and 34.0% in Palestine). Results indicate a statistically significant, larger proportion of current smokers in Lebanon (70.9%) compared with either Jordan (40.2%) or Palestine (36.8%). Though this pattern is somewhat reflected in current cigarette smoking (35.1% in Lebanon compared with 32.0% in Jordan and 28.2% in Palestine), the proportion of waterpipe smoking in Lebanon (39.5%) was more than three times that in Jordan (11.0%) and Palestine (12.9%). Consistent with prevalence rates of current waterpipe and cigarette smoking, participants in Lebanon stated larger purchases of all products compared with their counterparts in Jordan and Palestine (table 2).

**Table 1:** Sample characteristics

	Lebanon (N=1680) n (%)	Jordan (N=1925) n (%)	Palestine (N=1679) n (%)
Sex			
Male	840 (50.0)	1067 (55.4)	839 (50.0)
Female	840 (50.0)	858 (44.6)	840 (50.0)
Age, in years			
25 or less	296 (17.6)	340 (17.7)	299 (17.8)
26–35	377 (22.4)	440 (22.9)	411 (24.5)
36–45	375 (22.3)	456 (23.7)	344 (20.5)
46–55	367 (21.9)	370 (19.2)	363 (21.6)
56 or more	265 (15.8)	319 (16.6)	262 (15.6)
Monthly household income*			
1st quartile (lowest)	137 (8.2)	434 (22.6)	388 (23.1)
2nd quartile	647 (38.5)	731 (38.0)	699 (41.6)
3rd quartile	643 (38.3)	459 (23.8)	296 (17.6)
4th quartile (highest)	226 (13.5)	251 (13.0)	244 (14.5)
Don’t know/refuse to answer	27 (1.6)	50 (2.6)	52 (3.1)
Marital status			
Ever married	1433 (85.3)	1514 (78.7)	1425 (84.9)
Never married	247 (14.7)	411 (21.4)	254 (15.2)
Employment			
Full-time employed	748 (44.5)	663 (34.4)	570 (34.0)
Part-time employed	138 (8.2)	217 (11.3)	166 (9.9)
Other	794 (47.3)	1045 (54.3)	943 (56.2)
Education			
Middle school or less	607 (36.1)	1197 (62.2)	1295 (77.1)
High school/equivalent education	726 (43.2)	247 (12.8)	113 (6.7)
Graduate education	347 (20.7)	481 (25.0)	271 (16.1)
Nationality			
National of survey country	1610 (95.8)	1739 (90.3)	1631 (97.1)
Not a national of survey country	70 (4.2)	186 (9.7)	48 (2.9)
Role in daily household purchases			
Solely/jointly responsible	1195 (71.1)	1485 (77.1)	1153 (68.7)
Not responsible	485 (28.9)	440 (22.9)	526 (31.3)
Current cigarette smoker			
Yes	589 (35.1)	616 (32.0)	474 (28.2)
No	1091 (64.9)	1309 (68.0)	1205 (71.8)
Current waterpipe smoker			
Yes	663 (39.5)	211 (11.0)	216 (12.9)
No	1017 (60.5)	1714 (89.0)	1463 (87.1)
Current any tobacco smoker (cigarette and/or waterpipe)			
Yes	1191 (70.9)	774 (40.2)	617 (36.8)
No	489 (29.1)	1151 (59.8)	1062 (63.3)

*Monthly household income categories (in US dollars). Lebanon: (1) <$530; (2) $530–<$994; (3) $994–<$1987; (4) $1987 or more. Jordan: (1) <$423; (2) $423–<$705; (3) $705–<$1269; (4) $1269 or more. Palestine: (1) <$610; (2) $610–<$1068; (3) $1068–<$1525; (4) $1525 and more.

**Table 2:** Stated purchases of tobacco products, standardised to quantities per month among all respondents

	Lebanon (N=13 440)		Jordan (N=15 400)		Palestine (N=13 432)	
Tobacco product	Mean	% Null	Mean	% Null	Mean	% Null
Premium cigarettes	4.98	89.6	3.90	90.7	3.67	92.8
Discount cigarettes	14.24	75.6	6.11	86.4	1.85	96.4
Roll-your-own cigarettes	–	–	–	–	0.56	99.5
Premium waterpipe tobacco	7.16	83.6	1.03	96.6	1.13	97.0
Discount waterpipe tobacco	6.30	86.3	0.54	97.6	0.11	99.4
Non-flavoured waterpipe tobacco	0.81	98.6	0.02	99.5	–	–
Waterpipe tobacco home delivery	1.89	94.6	0.01	99.5	0.08	99.5
Premium waterpipe café	0.40	89.4	0.08	98.4	0.12	97.7
Discount waterpipe café	0.45	90.1	0.16	98.1	0.21	96.2

Roll-your-own cigarettes were only assessed in Palestine, and non-flavoured waterpipe tobacco was not assessed in Palestine.

### Own-price elasticities

All own-price elasticities for cigarette products were non-zero (statistically significant), except for roll-your-own cigarettes in Palestine (table 3). Elasticities for premium cigarettes were comparable across all countries and were roughly unitary, indicating that a change in the price of one product results in a change of comparable percentage (and opposite direction) in its demand. On the other hand, own-price elasticity for discount cigarettes was considerably higher in absolute value for Palestine compared with Lebanon and Jordan. In Lebanon, own-price elasticities of demand (in absolute value) for waterpipe products were higher than in the other two countries, except for the little-consumed traditional non-flavoured type. These elasticities ranged between −1.7 for discount waterpipe products and −2.3 for waterpipe consumed in premium cafés. In Jordan, the demand for waterpipe products was generally inelastic, whereby elasticities ranged between −0.3 for waterpipe smoking in discount cafés and −0.9 for discount waterpipe tobacco smoked in the home. Home-delivered waterpipe elasticity was not significant and that for non-flavoured waterpipe tobacco was statistically significant and positive (0.8). In Palestine, elasticities where significant were unitary at most (eg, premium waterpipe in cafés, −1.1) and generally pointed to an inelastic demand for waterpipe products (eg, discount waterpipe tobacco and discount waterpipe smoking in cafés, with elasticities of −0.6 and −0.3, respectively). Though premium waterpipe tobacco elasticity was positive, its relatively small magnitude (0.2) and its significance only at the 10% level undermine its robustness.

**Table 3:** Own-price elasticity estimates, by product and country

Tobacco product	Lebanon	Jordan	Palestine
Premium cigarettes	−1.157***	−1.080***	−1.042***
Discount cigarettes	−0.639***	−0.719***	−1.209***
Roll-your-own cigarettes	–	–	−0.065
Premium waterpipe tobacco	−1.949***	−0.601***	0.196*
Discount waterpipe tobacco	−1.700***	−0.915***	−0.650**
Non-flavoured waterpipe tobacco	0.095	0.816***	–
Waterpipe home delivery	−1.869***	0.104	−0.379
Premium waterpipe café	−2.312***	−0.674***	−1.120***
Discount waterpipe café	−1.699***	−0.335**	−0.291**

***p<0.01; **p<0.05; *p<0.10.

### Cross-price elasticities

Cross-price elasticities of premium versus discount cigarettes were highly significant in all three countries (table 4), though they were positive (ie, substitutes) in Lebanon and Jordan (0.2 and 0.3, respectively) and negative (ie, complements) in Palestine (−0.6). Premium cigarettes versus premium waterpipe tobacco elasticities were only significant in Jordan (−0.2), and premium cigarettes versus discount waterpipe tobacco elasticities were only significant in Lebanon (−0.1). Premium waterpipe tobacco versus discount waterpipe tobacco elasticities were significant and positive in Lebanon (0.5) and Jordan (0.5), whereas premium waterpipe tobacco versus non-flavoured waterpipe tobacco elasticities were only significant in Lebanon (−0.4). Finally, premium waterpipe tobacco versus waterpipe tobacco home delivery elasticities were significant and positive in Lebanon (0.2) and Palestine (0.5). Full results of all cross-price elasticity estimates are available in online supplemental tables.

10.1136/tobaccocontrol-2021-056616.supp1 Supplementary data

**Table 4:** Cross-price elasticity estimates, by product and country

Tobacco product	Lebanon	Jordan	Palestine
Premium cigarettes×discount cigarettes	0.166***	0.268***	−0.644***
Discount cigarettes×premium cigarettes	0.069	0.109*	−0.389***
Premium cigarettes×premium waterpipe tobacco	−0.004	−0.254***	−0.064
Premium waterpipe tobacco×premium cigarettes	−0.002	−0.038	0.292***
Premium cigarettes×discount waterpipe tobacco	−0.113***	0.101	0.224
Discount waterpipe tobacco×premium cigarettes	0.074	0.074	−0.067
Premium waterpipe tobacco×discount waterpipe tobacco	0.500***	0.504***	0.335
Discount waterpipe tobacco×premium waterpipe tobacco	0.209***	0.018	−0.237*
Premium waterpipe tobacco×non-flavoured waterpipe tobacco	−0.406***	−0.125	–
Non-flavoured waterpipe tobacco×premium waterpipe tobacco	−0.096	−0.136	–
Premium waterpipe tobacco×waterpipe home delivery	0.229***	0.403	0.476*
Waterpipe home delivery×premium waterpipe tobacco	−0.213***	−0.033	0.043
Premium waterpipe tobacco×premium waterpipe café	−0.063	−0.177	0.042
Premium waterpipe café×premium waterpipe tobacco	0.081**	0.112	0.335**
Premium waterpipe tobacco×discount waterpipe café	0.048	0.098	0.117
Discount waterpipe café×premium waterpipe tobacco	0.162***	0.161	−0.321**

***p<0.01; **p<0.05; *p<0.10.

## Discussion

To our knowledge, this study is the first to estimate own-price and cross-price elasticities of demand for both cigarettes and waterpipe tobacco products based on individual-level consumer data in the Eastern Mediterranean region. The study analysed data from large, nationally representative household surveys of smoking in Lebanon, Jordan and Palestine, and used a volumetric choice experiment to robustly and simultaneously evaluate the demand for eight different tobacco product varieties consumed in each country. Prior estimates of elasticity from Lebanon relied on household-level data that excluded waterpipe smoking in commercial establishments, and prior estimates from Jordan did not include waterpipe smoking. To our knowledge, no research has been published to date from Palestine on the economics of tobacco control. Given the common pattern of tobacco consumption across many countries in the Eastern Mediterranean region, it is expected that these estimates will also be informative to policymakers in other Eastern Mediterranean countries.

This study serves as an important contribution to the scarce literature on the elasticity of smoking in the Eastern Mediterranean region. Although price elasticity of smoking has been previously estimated in Lebanon and Jordan, the present study comprehensively assesses the own-price and cross-price elasticities of multiple tobacco product categories, based on nationally representative household samples, using robust and uniform methodologies across Lebanon, Jordan and Palestine. Broadly, we found the demand for premium cigarettes to be price elastic (around −1) across all three countries, while the demand for discount cigarettes was less elastic than premium cigarettes in Lebanon and Jordan and more elastic in Palestine. The demand for waterpipe tobacco was highly elastic in Lebanon, moderately elastic in Jordan, and inelastic in Palestine. The cross-price elasticity between cigarettes and waterpipe tobacco was near zero, suggesting that the two products are not considered to be close substitutes by consumers.

Compared with previous estimates, we found the elasticity for premium cigarettes in Lebanon to be higher (−1.2) compared with that of imported (ie, typically premium) cigarettes (−0.2), and that of discount cigarettes (−0.6) to be lower than the reported elasticity for local (ie, typically discount) cigarettes in the previous study (−1.5).^10^ Additionally, the higher elasticity for premium cigarettes in Lebanon, compared with previous estimates, may be attributable to price increases over the past decade and lower affordability over time. Meanwhile, we found the elasticity of premium waterpipe tobacco in the present study (−1.4) to be similar to the elasticity of waterpipe tobacco (−1.9) in the previous study. Also, cross-price elasticity estimates between waterpipe tobacco and cigarettes were close to zero, consistent with the previous study in Lebanon.

As for Jordan, the previous estimate of the elasticity of cigarettes (−0.6)^15^ was similar to our current estimate for discount cigarettes (−0.7) but lower than our estimate for premium cigarettes (−1.1). The previous study from Jordan did not differentiate between premium and discount cigarettes.^15^ Though our estimates are broadly consistent with previous estimates, differences may be attributable to methodology and temporal contexts. For example, stated preferences may yield higher elasticities than revealed preferences due to the hypothetical nature of scenarios in choice experiments.

To our knowledge, this study is unique in evaluating the price elasticity of demand for waterpipe smoking in the café setting, recognising the unique contextual factors surrounding waterpipe consumption needed to inform regulatory strategies.^13^ We found that the elasticities for waterpipe café smoking sessions were generally similar to the elasticities of store-purchased waterpipe tobacco, with two notable exceptions—discount waterpipe tobacco and café smoking in Jordan, and premium waterpipe tobacco and café smoking in Palestine. In addition, it is noteworthy that the cross-price elasticities between store-purchased waterpipe tobacco and café smoking sessions was weak and inconsistent, suggesting that smoking waterpipe in the home is not a close substitute to café smoking. This finding reinforces the notion that the demand for café smoking is partially explained by a social component, beyond the individual determinants of demand for tobacco smoking.^14^ It also underscores the need to extend price regulations to waterpipe-serving establishments as part of an overall comprehensive strategy for tobacco control.

Overall, the price elasticity of demand for tobacco products in Jordan and Palestine were comparable to the cigarette price elasticities reported in the literature, which cluster around the range of −0.2 to −0.6.^29^ However, price elasticities were markedly higher in Lebanon across several tobacco product categories. The exceptionally high elasticities of demand for tobacco products in Lebanon can be potentially explained with the high prevalence rates of cigarette and waterpipe smoking, as well as the survey administration coinciding with the early stages of a severe financial crisis that has since then diminished purchasing power in the country.

Comprehensive estimates of the cost of smoking are important for documenting the economic burden of tobacco use, designing effective tobacco control programmes, and identifying the healthcare needs of vulnerable populations. Even in Eastern Mediterranean countries where data are limited, estimates using available data, such as those described in the WHO toolkit for assessing economic costs,^30^ can be useful in advancing tobacco control. Our study demonstrates the need for tax policies in Eastern Mediterranean region countries to consider both cigarettes and waterpipe products, given the high prevalence of both tobacco products and their demonstrated elastic demand.

One key strength of this study was the use of choice experiments, and volumetric choice experiments in particular, which represent a robust methodology for providing premarket testing evidence for recommending fiscal policies. The tobacco industry itself has publicly asserted choice experiments as the standard for simultaneous evaluation of the effectives of tobacco product attributes on consumer choice.^31^ Choice experiments offer potentially powerful premarket testing evidence for recommending public health regulatory strategies. Whereas choice experiments are increasingly being applied to tobacco control research,^18^ to our knowledge, this is the first study to use volumetric choice experiments in tobacco control.

Despite the robustness of results in this study, several limitations are worth noting. Although choice experiments estimate choice behaviour, they may be an imperfect predictor of behaviour, especially when there are barriers to the behaviour. For example, cravings are common among users of all nicotine-containing tobacco products and social drivers for smoking can offer strong cues to influence the demand for smoking, especially in the case of waterpipe smoking, which is often consumed in social settings. Nevertheless, behavioural intention has repeatedly been shown to be a significant predictor of future behaviour. The estimated elasticities are average elasticities and hold for average prices.^32^ It is possible that much higher taxes, producing large increases in prices would produce larger effects on quantities than those predicted in this study. In other words, it is possible that the greater the increase in prices, the higher the price elasticity (in absolute values) and, thus, the larger the effect on quantities. However, we are unable to extrapolate exactly how elasticities would change when prices change outside of the ranges observed in this study.

## Conclusions

In conclusion, our results suggest that raising tobacco taxes could reduce tobacco use in the Eastern Mediterranean region. This study offers robust information on the economic relationship between cigarette and waterpipe smoking in countries where both products are highly prevalent. The findings of the current study can inform fiscal policies with the potential to contribute to reductions in the dual epidemic of cigarette and waterpipe smoking, while increasing government revenues. In particular, the findings suggest that governments should increase taxes on each product, and that tax policies will be effective because tobacco users would not substitute cigarettes with waterpipe tobacco.

**What this paper adds**

- Demand elasticities for cigarettes and more specifically for waterpipe tobacco in the Eastern Mediterranean region are not well researched.
- This research reports that price elasticity of demand for premium cigarettes is elastic (approximately −1) across Lebanon, Jordan and Palestine. The price elasticity of demand for premium waterpipe tobacco is highly elastic in Lebanon, moderately elastic in Jordan and inelastic in Palestine. The cross-price elasticities between cigarettes and waterpipe tobacco are relatively modest, suggesting that the two tobacco products are not close substitutes.
- This study provides timely policy relevant data to evaluate the potential effects of taxes on tobacco consumption, estimate revenue and predict substitution between cigarettes and waterpipe tobacco in response to price increases in the three countries.

## Data Availability

Data are available upon reasonable request.
