# Supplementary material for: Own-price and cross-price elasticities of demand for cigarettes and waterpipe tobacco in three Eastern Mediterranean countries: a volumetric choice experiment
Source: Tob Control. 2021 Jun 30;32(1):86–92. doi: 10.1136/tobaccocontrol-2021-056616 (PMC9763177; doi:10.1136/tobaccocontrol-2021-056616)
Supplement: Supplementary data [file tobaccocontrol-2021-056616supp001.pdf]

Supplementary Table 1. Cigarette-cigarette cross-price elasticity estimates

Tobacco product	Lebanon	Jordan	Palestine
Premium cigarettes			
Discount cigarettes	0.166***	0.268***	-0.644***
Roll your cigarettes (50g)	-	-	0.099
Discount cigarettes			
Premium cigarettes	0.069	0.109*	-0.389***
Roll your cigarettes (50g)	-	-	0.395***
Roll your cigarettes (50g)			
Premium cigarettes	-	-	0.443***
Discount cigarettes	-	-	0.071

*** p < .01; ** p < .05; * p <0.10.

Supplementary Table 2. Cigarette-waterpipe cross-price elasticity estimates

Tobacco product	Lebanon	Jordan	Palestine
Premium cigarettes			
Premium waterpipe tobacco	-0.004	-0.254***	-0.064
Discount waterpipe tobacco	-0.113***	0.101	0.224
Non-flavored waterpipe tobacco	-0.097	0.700**	-
Waterpipe tobacco home delivery	0.170**	0.184	-0.277
Premium waterpipe café	0.033	-0.165	-0.286**
Discount waterpipe café	0.028	-0.048	0.031
Discount cigarettes			
Premium waterpipe tobacco	-0.216***	0.184*	0.083
Discount waterpipe tobacco	0.030	-0.329**	-0.188
Non-flavored waterpipe tobacco	0.398***	-0.002	-
Waterpipe tobacco home delivery	0.044	-0.141	0.098
Premium waterpipe café	-0.026	-0.046	0.109
Discount waterpipe café	-0.107*	0.109	-0.004
Roll-your-own cigarettes			
Premium waterpipe tobacco	-	-	-0.768***
Discount waterpipe tobacco	-	-	0.150
Non-flavored waterpipe tobacco	-	-	-
Waterpipe tobacco home delivery	-	-	-0.447
Premium waterpipe café	-	-	0.226
Discount waterpipe café	-	-	-0.085

*** p < .01; ** p < .05; * p <0.10.

Supplementary Table 3. Waterpipe-cigarette cross-price elasticity estimates

Tobacco product	Lebanon	Jordan	Palestine
Premium waterpipe tobacco			
Premium cigarettes	-0.002	-0.038	0.292***
Discount cigarettes	-0.081**	0.181***	0.751***
Roll-your-own cigarettes	-	-	0.452***
Discount waterpipe tobacco			
Premium cigarettes	0.074	0.074	-0.067
Discount cigarettes	0.012	-0.060	-0.121
Roll-your-own cigarettes	-	-	0.141
Non-flavored waterpipe tobacco			
Premium cigarettes	0.004	-0.064	-
Discount cigarettes	0.015	0.059	-
Roll-your-own cigarettes	-	-	-
Waterpipe tobacco home delivery			
Premium cigarettes	0.016	-0.008	0.032
Discount cigarettes	0.050*	-0.027	-0.127
Roll-your-own cigarettes	-	-	-0.089
Premium waterpipe café			
Premium cigarettes	0.020	-0.006	-0.071
Discount cigarettes	-0.054*	0.012	0.090
Roll-your-own cigarettes	-	-	-0.427**
Discount waterpipe café			
Premium cigarettes	0.077	0.046	0.039
Discount cigarettes	-0.027	-0.084**	-0.225**
Roll-your-own cigarettes	-	-	0.155

*** p < .01; ** p < .05; * p <0.10.

Supplementary Table 4. Waterpipe-waterpipe cross-price elasticity estimates

Tobacco product	Lebanon	Jordan	Palestine
Premium waterpipe tobacco			
Discount waterpipe tobacco	0.500***	0.504***	0.335
Non-flavored waterpipe tobacco	-0.406***	-0.125	-
Waterpipe tobacco home delivery	0.229***	0.403	0.476*
Premium waterpipe café	-0.063	-0.177	0.042
Discount waterpipe café	0.048	0.098	0.117
Discount waterpipe tobacco			
Premium waterpipe tobacco	0.209***	0.018	-0.237*
Non-flavored waterpipe tobacco	-0.096	-0.136	-
Waterpipe tobacco home delivery	-0.023	0.185	-0.304
Premium waterpipe café	0.027	0.072	0.143
Discount waterpipe café	0.065	0.097	0.098
Non-flavored waterpipe tobacco			
Premium waterpipe tobacco	-0.074*	0.184*	-
Discount waterpipe tobacco	-0.008	-0.009	-
Waterpipe tobacco home delivery	0.197***	0.215	-
Premium waterpipe café	-0.022	0.245	-
Discount waterpipe café	-0.063	-0.134	-
Waterpipe tobacco home delivery			
Premium waterpipe tobacco	-0.213***	-0.033	0.043
Discount waterpipe tobacco	0.137***	0.126	-0.242
Non-flavored waterpipe tobacco	0.377***	-0.022	-
Premium waterpipe café	-0.043	-0.240	0.410***
Discount waterpipe café	-0.045	0.036	0.243**
Premium waterpipe café			
Premium waterpipe tobacco	0.081**	0.112	0.335**
Discount waterpipe tobacco	-0.012	-0.225*	0.485
Non-flavored waterpipe tobacco	0.010	0.188	-
Waterpipe tobacco home delivery	-0.288***	0.079	0.763**
Discount waterpipe café	0.226***	0.171	-0.177
Discount waterpipe café			
Premium waterpipe tobacco	0.162***	-0.073	-0.207
Discount waterpipe tobacco	-0.241***	0.079	-0.236
Non-flavored waterpipe tobacco	-0.064	-0.345	-
Waterpipe tobacco home delivery	0.049	-0.384	-0.097
Premium waterpipe café	0.157***	0.161	-0.321**

*** p < .01; ** p < .05; * p <0.10.
